# Acupuncture therapy for drug addiction

**DOI:** 10.1186/s13020-016-0088-7

**Published:** 2016-04-05

**Authors:** Farid Esmaeili Motlagh, Fatimah Ibrahim, Rusdi Abd Rashid, Tahereh Seghatoleslam, Hussain Habil

**Affiliations:** Department of Biomedical Engineering, Faculty of Engineering, University of Malaya, 50603 Kuala Lumpur, Malaysia; Centre for Innovation in Medical Engineering, Faculty of Engineering, University of Malaya, 50603 Kuala Lumpur, Malaysia; Centre of Addiction Sciences, University of Malaya, 21st Floor, Wisma Research and Development, Jalan Pantai Baru, 59200 Kuala Lumpur, Malaysia; Shahid Beheshti University of Medical Sciences, Tehran, Iran

## Abstract

Acupuncture therapy has been used to treat substance abuse. This study aims to review experimental studies examining the effects of acupuncture on addiction. Research and review articles on acupuncture treatment of substance abuse published between January 2000 and September 2014 were searched using the databases ISI Web of Science Core Collection and EBSCO’s MEDLINE Complete. Clinical trial studies on the efficacy of acupuncture therapy for substance abuse were classified according to substance (cocaine, opioid, nicotine, and alcohol), and their treatment protocols, assessments, and findings were examined. A total of 119 studies were identified, of which 85 research articles addressed the efficacy of acupuncture for treating addiction. There were substantial variations in study protocols, particularly regarding treatment duration, frequency of electroacupuncture, duration of stimulation, and choice of acupoints. Contradictory results, intergroup differences, variation in sample sizes, and acupuncture placebo effects made it difficult to evaluate acupuncture effectiveness in drug addiction treatment. This review also identified a lack of rigorous study design, such as control of confounding variables by incorporating sham controls, sufficient sample sizes, reliable assessments, and adequately replicated experiments.

## Background

In 1997, the National Institutes of Health accepted acupuncture therapy as an acceptable procedure complementary to Western medicine [1]. Evidence for its therapeutic effects comes mainly from clinical practice and research into pain control, fibromyalgia, headaches, Parkinson’s disease, schizophrenia, and depression [2]. Acupuncture therapy can be administered using either manual insertion of needles or electroacupuncture (EA), a mild electrical stimulation of acupoints. Extended acupuncture methods may involve finger pressure (acupressure) and laser therapy [3].

In 1985, Dr. M. Smith finalized the National Acupuncture Detoxification Association (NADA) protocol that is currently practiced in over 250 hospitals in the United Kingdom and United States [4]. In 1996, the World Health Organization accepted acupuncture as a treatment for drug abuse [5]. The latest modification to this treatment protocol was developed in 2005 by Dr. Ji Sheng from Peking University, Beijing, China [6]. Currently, more than 700 addiction treatment centers use acupuncture as an adjunctive procedure [7].

Prominent effects of acupuncture are increases in the levels of enkephalin, epinephrine, endorphin, serotonin, norepinephrine, and dopamine in the central nervous system and plasma [8] that might mediate substance abuse. Acupuncture has been used to treat addiction for three decades [2–89]. For example, auricular acupuncture (AA) is effective in treating alcohol and drug abuse in both Europe and the United States [4].

However, several clinical trials have indicated that acupuncture was not effective in treating addiction [2, 3, 31, 67, 69, 78–80]. Thus, the efficacy of the NADA protocol has been reassessed over the last decade [7]. Several factors have been studied to evaluate the efficacy of acupuncture therapy; for example, treatment protocol, choice of acupoints, duration of acupuncture, study design diversity, sample size, addiction history, and assessment techniques.

This study aims to review the published research on acupuncture therapy for substance abuse in relation to study type, authors, funding agencies, countries, agonist substances, and acupoints used for stimulation. Experimental studies published between January 2000 and September 2014 were systematically reviewed and analyzed to try to resolve the lack of agreement about acupuncture’s efficacy for substance abuse.

## Review

### Literature search

**Fig. 1:**
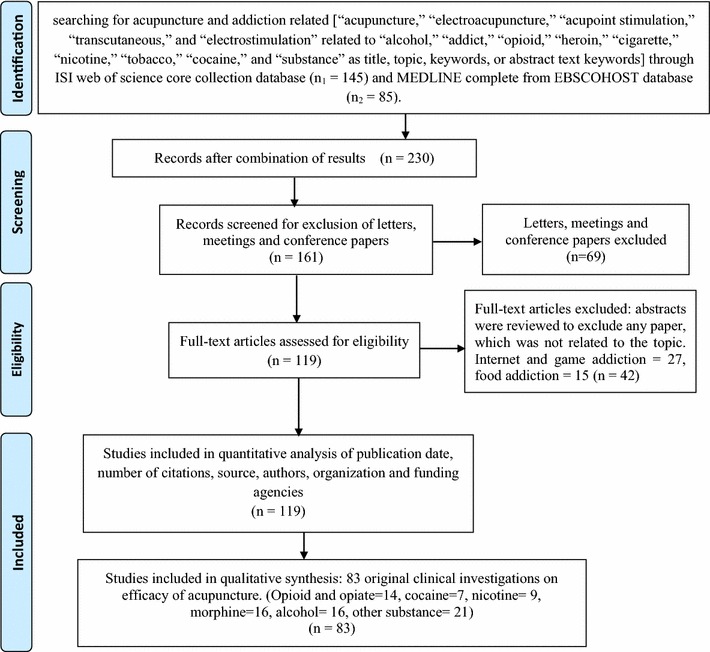
Flow chart of selection process

A search of the ISI Web of Science Core Collection and EBSCOHost (MEDLINE Complete) databases for the period January 2000 to September 2014 was conducted to identify acupuncture clinical trials. Keywords, topics available in the databases, and titles were searched for the following terms: “acupuncture,” “electroacupuncture,” “acupoint stimulation,” “transcutaneous,” and “electrostimulation” as single words or combinations (total number of articles: 25 358). The results were refined to exclude non-English language materials. The preliminary findings of the first phase were refined by several parallel filters to identify documents relevant to acupuncture treatment of substance abuse. The operator between the filters was the “OR” command. Single, relevant words were selected for each filter and included any combination of the following: “alcohol,” “addict,” “opioid,” “heroin,” “cigarette,” “nicotine,” “tobacco,” “cocaine,” and “substance” as title, topic, keywords, or abstract text (total number of articles: 230) from both searched databases. The search results were collated and filtered to exclude proceedings papers and letters, yielding 161 studies. The abstracts of these documents were reviewed to exclude papers related to other addiction fields such as the Internet, food, or games; 119 documents comprised the refined, selected results. Three authors (FEM, RR, and TS) independently assessed studies for eligibility and crosschecked the material for study relevance. The publication selection process was shown in Fig. 1.

The articles were classified according to their specifications, including publication date, number of citations, source, authors, organization, and funding agencies. All original research papers were examined for their efficacy and method of treating different categories of addiction to agonist substances (e.g., cocaine, opioid and opiate, nicotine, alcohol, morphine). Original clinical trials that investigated the efficacy of acupuncture therapy were divided into six categories based on substance dependence (cocaine, opioid and opiate, nicotine, alcohol, morphine, and other substances) with a narrative review of their methods and results. Although morphine is an opioid, it has been assigned its own section because of the high number of publications on this topic. Heroin, methadone, and opiates are discussed in the opioid section.

**Fig. 2:**
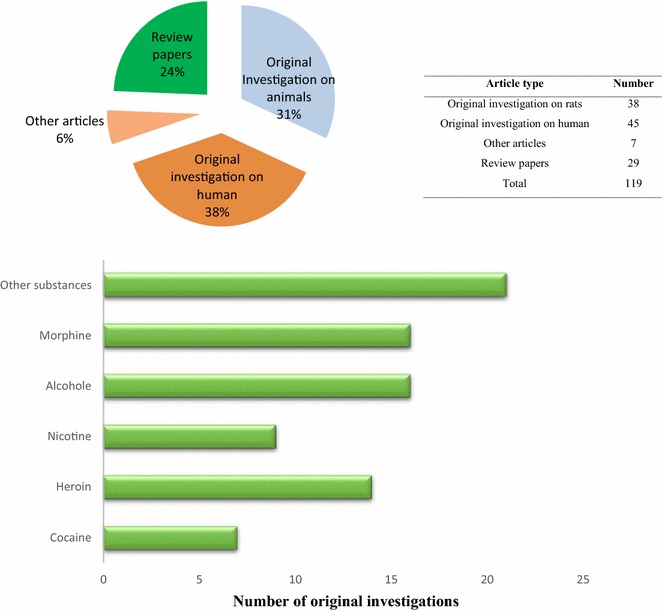
Classification of selected articles into review papers and original investigations on humans and animals. The lower diagram shows the number of original clinical investigations according to substance. Morphine and alcohol have the most articles. Each of the columns is described separately in Tables 2, 3, 4, 5, 6, 7

The findings are discussed and compared according to type of addictive substance. There were 96 articles and 29 review papers; 83 articles were original investigations (76 of which were clinical trials of acupuncture efficacy), with 45 articles involving human beings and 38 involving animals. Figure 2 shows the percentage of all documents in each type of category. Original investigations of humans and animals were classified separately according to type of substance dependence. These articles placed more of an investigative emphasis on morphine and alcohol than on other substances.

The total number of citations for all documents was 1495 (mean = 15.83 and standard deviation = 15.27, citation range 2–87 by excluding 29 articles cited zero times or only once). The top ten most cited articles were shown in Table 1. The United States (43 articles) and China (40 articles) published almost 70 % of all articles. Among the Asian countries, China and South Korea published 59 articles, comprising 50 % of publications. Their funding agencies were also the top supporters in this field. Peking, Kyung Hee, and Daegu Haany Universities were the top three organizations, publishing 40 articles since 2000.

**Table 1:** Top 10 most cited articles from 2000 to 2014

Title	First author	Source title	Publication year	Total citations
Acupuncture: An evidence-based review of the clinical literature [90]	Mayer DJ	Annual Review of Medicine	2000	87
A randomized controlled trial of auricular acupuncture for cocaine dependence [10]	Avants SK	Archives of Internal Medicine	2000	67
Acupuncture for the treatment of cocaine addiction—a randomized controlled trial [61]	Margolin A	Journal of the American Medical Association	2002	66
Clinical research on acupuncture: Part l. What have reviews of the efficacy and safety of acupuncture told us so far? [91]	Birch S	Journal of Alternative and Complementary Medicine	2004	64
Peripheral neuropathy: Pathogenic mechanisms and alternative therapies [92]	Head, Kathleen A	Alternative Medicine Review	2006	50
Acupuncture and related interventions for smoking cessation [78]	White AR	Cochrane Database of Systematic Reviews	2006	43
A large randomized placebo controlled study of auricular acupuncture for alcohol dependence [16]	Bullock ML	Journal of Substance Abuse Treatment	2002	41
Peripheral electric stimulation inhibits morphine-induced place preference in rats [75]	Wang B; Luo, F	NeuroReport	2000	39
Acupuncture in clinical neurology [67]	Rabinstein AA	Neurologist	2003	37
Traditional Chinese medicine in treatment of opiate addiction [93]	Shi, Jie	Acta Pharmacologica Sinica	2006	34

**Fig. 3:**
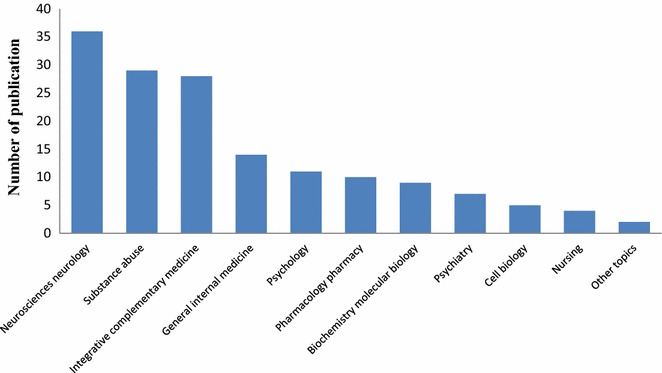
Number of research articles in each area. Neuroscience, substance abuse, and complementary medicine are ranked highest

**Fig. 4:**
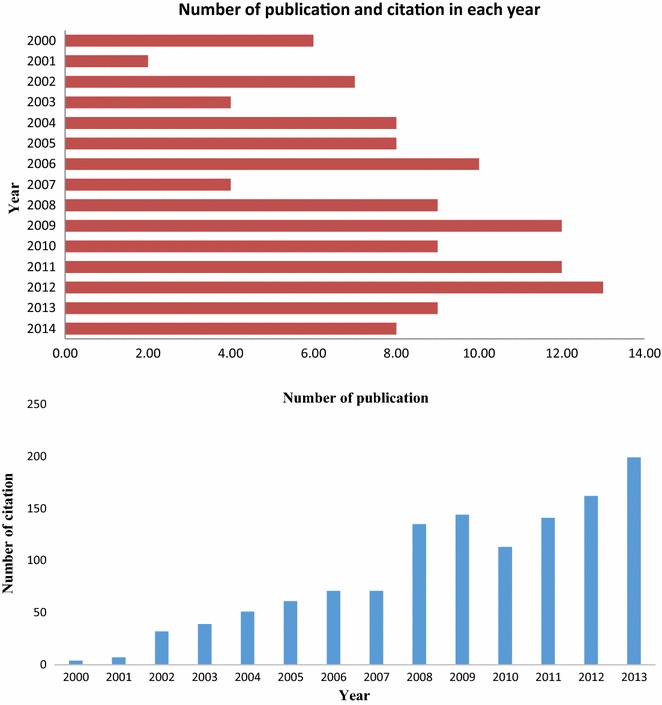
Number of publications (*top*) and citations (*bottom*) per year. This figure shows that since 2008, the number of citations has increased to 200 per year. Before 2008, it was about 50 per year

The published articles were associated with various research areas (Fig. 3). About 80 % of the articles focused on neuroscience and neurology, substance abuse, and integrative complementary medicine research areas. Published articles for each year were shown in Fig. 4.

Original experimental research articles were reviewed according to type of substance dependence (Fig. 2); the treatment method, subjects, objectives, and assessments of clinical trials for each group were shown in Tables 2, 3, 4, 5, 6, 7.

**Table 2:** Original investigations of acupuncture therapy effects in cocaine-dependent subjects

Publication year; first author		Objectives	Subjects	Stimulated acupoints (acupuncture type, acupoints, frequency)	Assessments	Outcomes
2000	Avants [10]	To evaluate the AA efficacy in cocaine addiction treatment compared to needle-insertion and no-needle relaxation control conditions	82 Cocaine-dependent, methadone-maintained patients	Auricular acupuncture at 4 NADA points (sympathetic, lung, liver, and *Shenmen* (HT7)), 5 times a week for 8 weeks	Urine toxicology screens 3-times-weekly	Acupuncture showed positive results compared to control groups for treatment of cocaine dependence
2002	Margolin [60]	To Compare two cocaine addiction clinical trials of AA to explore consistency of treatment effects	165 Cocaine-dependent, methadone-maintained patients (Study 1, n = 82; Study 2, n = 83)	Auricular acupuncture at 4 NADA points (sympathetic, lung, liver, and *Shenmen* (HT7)), 5 times a week for 8 weeks	Urine toxicology screens 3-times-weekly, retention in treatment, treatment attendance, treatment credibility, therapeutic alliance, and acute effects	The results of two groups were controversial and no conclusion could be made regarding the effectiveness of AA
2002	Margolin [61]	To evaluate the AA efficacy in cocaine addiction treatment	620 Cocaine-dependent methadone-maintained patients. 412 Cocaine only and 208 opiates + cocaine	Auricular acupuncture at 4 NADA points (sympathetic, lung, liver, and *Shenmen* (HT7)), 5 times a week for 8 weeks	Urine toxicology during treatment and at the 3- and 6-month post randomization follow-up, and retention in treatment	acupuncture was not more effective than a needle insertion or relaxation control in reducing cocaine use
2005	Margolin [59]	To evaluate effects of acupuncture and spirituality, therapy in the treatment of HIV-positive drug users	40 HIV-seropositive, cocaine-dependent, methadone-maintained patients	Auricular acupuncture at 5 NADA points, 5 times weekly for 8 weeks	Urine toxicology twice weekly, depression and anxiety at pre- and post-treatment	acupuncture and a spirituality-focused psychotherapy was effective in reducing the cocaine use
2009	Lee and Bombi [44]	To investigate the effects of acupuncture on the repeated cocaine-induced neuronal and behavioral sensitization alternations	32 Male Sprague–Dawley rats, n = 15 acupuncture	Acupuncture bilaterally at *Shenmen* (HT7) for 1 min	Cocaine-induced locomotor activity and the expression of tyrosine hydroxylase (TH) in the brain	acupuncture was effective for inhibiting the behavioral effects of cocaine by possible modulation of the central dopaminergic system
2012	Yoon [84]	To investigate the effects of acupuncture on cocaine-seeking and the expression of c-Fos and the transcription factor cAMP response element-binding protein (CREB)	24 Male Sprague–Dawley rats, n = 6 EA	Acupuncture at *Shenmen* (HT7) as study and *Yangxi* (LI5) as control for 1 min	Cocaine-seeking behavior, surface expression, and phosphorylated CREB (pCREB) activation in the NAc shell	acupuncture attenuated stress-induced relapse by regulating neuronal activation in the NAc shell
2013	Kim and Seol Ah [42]	To explore the peripheral mechanisms underlying acupuncture treatment for drug addiction	12 Male Sprague–Dawley rats	Acupuncture at *Shenmen* (HT7) as study and *Yangxi* (LI5) as control for 1 min	Suppression of cocaine-induced locomotor activity	acupuncture inhibited the cocaine-induced locomotor activity

Acupoints in NADA protocol are located at (sympathetic: in the deltoid fossa at the junction of the infra-antihelix crus and the medial order of the helix, lung: in the center of the cavum concha, liver: located in the posterior to upper portion of the helix crus, kidney: in the cleft between the upper plateau, and the helix)

### Cocaine

Avants and Margolin have evaluated the efficacy of AA for cocaine addiction treatment in four studies on human subjects. Although promising results were reported in their first study on 82 cocaine-dependent subjects [10], another study on 83 cocaine-dependent subjects found AA to be effective in reducing cocaine in only one of two trials [60]. When the original study was repeated with 620 subjects, no effect was found [61]. These researchers also conducted a study in 2005 on 40 cocaine abusers who had tested positive for the human immunodeficiency virus and were under methadone maintenance; no difference was found between the standard and reduced NADA protocols for cocaine use [59].

Three studies on rats were conducted to explore the effects of bilateral stimulation at the *Shenmen* (HT7) points. Modulation of the central dopaminergic system by acupuncture might be effective in preventing the behavioral effects of cocaine in rats [44]. By regulating neuronal activation in the nucleus accumbens (NAc) shell, acupuncture reduced stress-induced relapse [84]. The effect of acupuncture on the inhibition of cocaine-induced locomotor activity was mediated by A-fiber activation of the ulnar nerve in rats [42]. See Table 2 for study details.

### Opioids and opiates

In 2002, Montazeri investigated the efficacy of acupuncture at *Hegu* (LI4), *Neiguan* (PC6), *Shenmen* (HT7), *Taichong* (LR3), *Zusanli* (ST36), *Dazhui* (DU14), and *Baihui* (DU20) in 40 male adult heroin- or opium-addicted patients. The severity of withdrawal symptoms declined when acupuncture was used in rapid opiate detoxification [63]. Liu (2007) used functional magnetic resonance imaging to show that hypothalamus activation associated with manual acupuncture at *Zusanli* (ST36) was more robust in heroin addicts compared with healthy subjects [55]. EA (2 Hz) at *Zusanli* (ST36) and *Sanyinjiao* (SP6) was effective in reducing active responses elicited by discrete cues in rats [56]. The same EA treatment showed promise in treating heroin-seeking behaviors when combined with extinction therapy [33]. EA (2 Hz) at the same points—*Zusanli* (ST36) and *Sanyinjiao* (SP6)—activated the endogenous opioid cannabinoid and the dopamine systems in rats [81].

An evaluation of the event-related potentials of heroin addicts before and after acupuncture at *Neiguan* (PC6) and *Zusanli* (ST36) suggested that EA might potentially lower relapse rates by inhibiting attention bias to heroin [37]. The presentation of heroin cues could induce activation in craving-related brain regions, which are involved in reward, learning and memory, cognition, and emotion. Acupuncture at *Zusanli* (ST36) rapidly suppressed the activation of these specific brain regions related to craving [17]. Transcutaneous electric acupoint stimulation was a possible adjunctive treatment to pharmacological treatments for heroin detoxification [62]. Acupuncture at *Dazhui* (GV14) and *Baihui* (DU20) prevented brain cell apoptosis in heroin-readdicted rats, normalized neuronal ultrastructure in the ventral tegmental area of heroin relapse rats, and protected nerve cells against injury in heroin relapse rats [32, 88].

Recent studies of acupuncture’s effectiveness as an adjunct therapy in methadone maintenance programs have been controversial. In 2009, Bearn demonstrated a lack of effect for adjunctive methadone maintenance treatment with AA upon withdrawal severity or craving [11]. In 2013, Pei Lin showed a lack of AA effectiveness on the number of daily consumed cigarettes, relapse rate, and withdrawal symptoms, and examined patients’ satisfaction and coping with AA as an adjunct treatment to methadone maintenance treatment among Malaysian subjects [57, 58]. However, Chan et al. [22] claimed that 2 weeks of acupuncture therapy reduced the daily dose of methadone and was also associated with greater improvement in sleep latency. See Table 3 for study details.

**Table 3:** Original investigations of acupuncture therapy effects in opioid- and opiate-dependent subjects

Publication year, first author		Objectives	Subjects	Stimulated acupoints. (acupuncture type, acupoints, frequency)	Assessments	Outcomes
2002	Montazeri, Kamran [63]	To identify the effects of body acupuncture on rapid opiate detoxification	40 Opioid addicts during ROD by naloxone, n = 20 acupuncture	Bilaterally acupuncture at *Hegu* (LI4), *Neiguan* (PC6), *Shenmen (HT7)*, *Taichong* (LR3), *Zusanli* (ST36), *Baihui* (DU20) and *Dazhui* (DU14) 30 min for 3 days	Severity of withdrawal reaction (Clinical Institute Narcotic Assessment (CINA) score)	acupuncture reduced the severity of withdrawal symptoms associated with rapid opiate detoxification
2007	Liu [55]	To investigate the activation in the hypothalamus associated with acupuncture stimulation	Six healthy men and six heroin addicts	Acupuncture at *Zusanli* (ST36) with rotation for 11 min	fMRI, Cortisol level and psychophysical responses, including the deqi sensation, anxiety, and sharp pain	Acupuncture caused activation of the hypothalamus among addicts
2009	Bearn [11]	To investigate AA effects as an adjunct to MMT upon withdrawal severity or craving	83 Opioid users under MMT	Acupuncture at 5 points of cartilage ridge area in the ear, 30–40 min for 14 days	Daily measures of withdrawal severity and craving using the short opiate withdrawal scale and an eight-item craving questionnaire	AA had no effect upon withdrawal severity or craving when provided as an adjunct to a standard methadone detoxification treatment
2010	Meade [62]	To evaluate the effectiveness of TEAS as an adjunctive treatment for inpatients receiving opioid detoxification	48 Men and women under detoxification with buprenorphine-naloxone	2 and 100 Hz TEAS at *Hegu* (LI4) and *Neiguan* (PC6), 30 min daily for 4 days	The addiction severity index, opioid withdrawal scale, brief pain inventory, the Pittsburgh sleep quality index, physical and mental health status by the medical outcomes survey	TEAS was effective in using drugs and improving the pain interference and physical health
2011	Xia [81]	To investigate the rewarding effect of EA	68 Male Sprague–Dawley rats	2 Hz EA at *Neiguan* (PC6) and *Zusanli* (ST36), 30 min for 5 days	Conditioned place preference (CPP)	EA was capable of inducing CPP in the rat via the activation of the endogenous opioid-, cannabinoid- and dopamine-systems
2011	Jiang [37]	To compare the changes of cognitive attention-related brain function before and after EA	Ten Heroin addicts and ten healthy subjects	2 Hz bilateral EA at *Neiguan* (PC6) and *Zusanli* (ST36)	ERP at 64 electrode spots before and after EA intervention task on the positive emotional clue (PEG), negative emotional clues (NEC), and heroin-related clue (HRC). The P200 amplitude components on (Fz, Cz, and Pz)	Electroacupuncture effectively inhibited the attention bias to heroin
2012	Cai [17]	To understand the influence of heroin cue exposure on brain activation	12 Heroin addicts and 12 healthy subjects	non-twirled acupuncture and twirled acupuncture at bilateral *Zusanli* (ST36) for 1 min	fMRI during heroin cue exposure	Acupuncture can rapidly suppress the activation of specific brain regions related to craving as an intervention for drug craving
2012	Liu [56]	To investigate the beneficial effects of EA on heroin-seeking behavior	40 Male Sprague–Dawley rats, n = 10 EA	2 Hz EA at *Zusanli* (ST36) and *Sanyinjiao* (SP6), once a day for 14 days during heroin abstinence	Contextual and discrete cue-induced reinstatement of active responses. Fos-positive nuclei detection in the nucleus accumbens (NACc) core and shell	acupuncture was effective in preventing relapse to drug addiction
2013	Hu [33]	To investigate the effects of EA on the extinction of heroin-seeking behavior	18 Male Sprague–Dawley rats, n = 6 EA	2 Hz EA at *Zusanli* (ST36) and *Sanyinjiao* (SP6), once a day for 7 days	The extinction response of heroin associated cues and applied immunohistochemistry to detect FosB-positive nuclei in the nucleus accumbens core	Acupuncture enhanced extinction learning when combined with extinction therapy for the treatment of heroin addiction
2013	Lua, Pei Lin [57]	To find the effects of AA in number of consumed daily cigarettes, relapse rate, and withdrawal symptoms	40 MMT, 29 MMT + AA human subjects	Auricular acupuncture at 5 points NADA points, 3 times a week	Malay HOQOLBREF, withdrawal symptoms	Acupuncture adjunct to MMT was beneficial in managing addiction behaviors
2013	Lua, Pei Lin [58]	To examine the patients’ satisfaction and coping with AA as an adjunct treatment to MMT	40 MMT, 29 MMT + AA human subjects	Auricular acupuncture at 5 points NADA points, 3 times a week	Patient satisfaction with pharmaceutical care questionnaire (PSPCQ) and Malay brief COPE-27	Acupuncture adjunct to MMT did not influence patient satisfaction and their coping ways
2014	Chan [22]	To examine the effectiveness of acupuncture for heroin addicts on methadone maintenance	60 Heroin addicts in MMP	EA at *Hegu* (LI4), *Zusanli* (ST36) and *Shenmen (HT7),* twice a week for 4 weeks	Daily consumption of methadone, variations in the 36-item Short Form Health Survey-36 (SF-36) and the Pittsburgh Sleep Quality Index (PSQI) scores, and heroin craving	Acupuncture adjunct to MMT was useful in reducing the daily dose of methadone and great improvement in sleep latency at follow-up
2014	Hou [32]	To observe cell apoptosis in the hippocampus and frontal lobe of heroin readdicted rats by electron microscopy.	40 Wistar rats during the detoxificstion by methadone	Acupuncture at *Baihui* (GV20) and *Dazhui* (GV14), 30 min for 5 successive days	Morphology of nerve cells, Bax expression and Bcl-2 expression in the frontal cortex and hippocampus	Acupuncture was effective in preventing brain cell apoptosis in heroin readdicted rats
2014	Zhang [88]	To verify the relationship between acupuncture, neurotrophic factor expression and brain cell structural changes	32 Wistar rats, n = 16 acupuncture	Acupuncture at *Baihui* (DU20) and *Dazhui* (DU14) for 30 min, once a day for five consecutive days	The neuronal ultrastructure of the ventral tegmental area, brain-derived and glial cell line-derived neurotrophic factor expression in the ventral tegmental area	Acupuncture protected brain neurons against injury in rats with heroin relapse

Acupoints in NADA protocol are located at (sympathetic in the deltoid fossa at the junction of the infra-antihelix crus and the medial order of the helix, lung in the center of the cavum concha, liver located in the posterior to upper portion of the helix crus, kidney in the cleft between the upper plateau, and the helix)

### Nicotine

Acupuncture stimulation at *Zusanli* (ST36) exerted a therapeutic effect on nicotine detoxification [21] and acupuncture at *Zusanli* (ST36) or *Shenmen* (HT7) might attenuate anxiety-like behavior following nicotine withdrawal by modulating corticotrophin-releasing factor in the amygdala [20]. Smoking withdrawal symptoms could be ameliorated by acupuncture treatment [18]. In one study, acupuncture at *Shenmen* (HT7) attenuated cigarette withdrawal symptoms more than acupuncture at *Shousanli* (LI10) [19]. Real acupuncture (as opposed to sham acupuncture) at *Shenmen* (HT7) alleviated cue-induced cravings through the regulation of activity in brain regions (medial prefrontal cortex, premotor cortex, amygdala, hippocampus, and thalamus) related to craving scores in the initial abstinence phase [38].

However, one study failed to find any effect of acupuncture on cotinine serum levels, carbon monoxide exhalation, and smoking quit rate in 59 smokers [83]. It has been suggested that DRD2 gene TaqI A polymorphism was related to AA response in smoking cessation treatment [65]. Auricular transcutaneous electrical neurostimulation relieved withdrawal symptoms and decreased anxiety and stress levels during the detoxification period in a study of six smokers [15]. Auricular transcutaneous electrostimulation therapy might be an acceptable alternative therapy for smoking cessation [72]. See Table 4 for study details.

**Table 4:** Original investigations of acupuncture therapy effects in nicotine-dependent subjects

Publication year; first author		Objectives	Subjects	Stimulated acupoints (acupuncture type, acupoints, frequency)	Assessments	Outcomes
2004	Chae [21]	To investigate the acupuncture effects on the functional alterations of the mesolimbic dopaminergic systems	35 Male Sprague–Dawley rats	Acupuncture at *Zusanli* (ST36), *Shenmen* (HT7), or *Taiyuan* (LU9) for 4 days	Nicotine-induced FLI in the striatum and the nucleus accumbens	acupuncture had a therapeutic effect on nicotine addiction
2005	Park [65]	To examine whether the DRD2 TaqI A polymorphism is associated with the response to acupuncture	231 Healthy Korean male smokers	AA at lung, throat, *Shenmen* (HT7), and endocrine points for 96 s, 3 times for a week	Cigarette consumption, the desire to smoke, and Genomic DNA extracted from blood samples	Acupuncture was effective to influence the DRD2 TaqI A polymorphism
2008	Chae [20]	To investigate the effect of acupuncture on anxiety-like behavior and corticotrophin-releasing factor (CRF) and neuropeptide Y (NPY) mRNA expression in the amygdala during nicotine withdrawal	38 Male Sprague–Dawley rats, n = 18 acupuncture	Acupuncture at *Zusanli* (ST36), *Shenmen* (HT7), 30 s for 3 days	The anxiogenic response by using an elevated plus maze. CRF and NPY mRNA levels by using reverse transcription polymerase chain reaction (RT-PCR) analysis	acupuncture attenuated anxiety-like behavior following nicotine withdrawal
2008	Bonnette [15]	To explore the effects of ATENS in combination with addiction education, behavioral training and coaching	6 Smokers	Acupuncture at 5 NADA protocol or 1-3 points, 5 times a week for 8 weeks	In-depth interviews for withdrawal symptoms, anxiety and stress levels	auriculotherapy relieved withdrawal symptoms and reduced anxiety and stress levels during the detoxification
2009	Yeh [83]	To evaluate the effects of a 6-week acupoint stimulation program for quitting	59 Smokers	Acuouncture at *Shenmen* (HT7), lung, stomach, mouth and endocrine and *Tim mee for* 20 min, once a week for 6 weeks	Demographic factors, serum cotinine, carbon monoxide exhalation, daily tobacco consumption, and quit smoking rate of participants before and after the 6-week intervention	Acupuncture showed no statistically significant effect on quitting smoking
2010	Thanavaro [72]	To explore the efficacy of ATET as an adjunctive treatment to intensive individual counseling on smoking cessation	29 Subjects	Auricular transcutaneous electrostimulation therapy at 10 areas on the pinna	The “Fagerstrom Test for Nicotine Dependence,” the “What Are Your Triggers Test” and the “Why Do I Smoke Quiz.”	individual counseling may produce smoking cessation rates comparable to counseling with pharmacotherapy
2010	Chae [18]	To investigate the effect of acupuncture on the selective attention to smoking-related visual cues	29 Smokers	Acupuncture, *Shenmen* (HT7), (NA)	The attentional bias and cigarette withdrawal scale	Acupuncture ameliorated the smoking withdrawal symptoms as well as the selective attention to smoking-related visual cues
2011	Chae [19]	To investigate effects of acupuncture on ameliorating cigarette withdrawal symptoms	29 Smokers, n = 15 acupuncture	Acupuncture at *Shenmen* (HT7) or *Shousanli* (LI10), 20 min for 3 days	The cigarette withdrawal scale (CWS), comparing the low-frequency/high-frequency (HF/LF) ratio in the HRV of the RA and SA groups	acupuncture attenuated withdrawal symptoms and smoking cues-induced autonomic responses
2013	Kang [38]	To investigate acupuncture effects on ameliorating cravings induced by smoking-related visual cues	25 Male smokers	Acupuncture at *Shenmen* (HT7) for 1 min	fMRI and craving scores to smoking-related visual cues were assessed before and after RA or sham treatment	acupuncture alleviated cue-induced cravings through the regulation of activity in brain regions involved in attention, motivation, and reward

Acupoints in NADA protocol are located at (sympathetic: in the deltoid fossa at the junction of the infra-antihelix crus and the medial order of the helix, lung: in the center of the cavum concha, liver: located in the posterior to upper portion of the helix crus, kidney: in the cleft between the upper plateau, and the helix)

### Alcohol

Conflicting results from two large randomized single-blind, placebo-controlled trials suggested that acupuncture was not effective in reducing alcohol use [16, 39]. However, promising results have been found using acupuncture as an adjunctive treatment to carbamazepine medication to reduce the severity of alcohol withdrawal symptoms [39]. In one study, AA failed to reduce the duration and severity of alcohol withdrawal symptoms [43]; another study found no advantage for laser AA in treating alcohol withdrawal [74]. However, research indicated that laser therapy helps to promote the release of endorphins in the body and decreases discomfort accompanying alcohol withdrawal [87]. It might therefore be a safe and painless beneficial adjunct treatment for alcoholism [87].

Acupuncture at *Zusanli* (ST36) or *Sanyinjiao* (SP6) modulated postsynaptic neural activation in the striatum and NAc in rats [89]. Acupuncture at *Shenmen* (HT7) normalized dopamine release in the mesolimbic system [89], modulated mesolimbic dopamine release, and suppressed the reinforcing effects of ethanol [82]. Activation of the endogenous opiate system might be responsible for *Zusanli* (ST36) and *Sanyinjiao* (SP6) stimulation effects on alcohol intake in alcohol-dependent rats [64].

EA applied at *Zusanli* (ST36) was more effective than EA at *Shenshu* (BL23) at normalizing alcohol-drinking behavior in rats [86]; the activity of serotonergic neurons in the reward system pathway of the brain might be increased and prolonged by acupuncture [85]. EA at the combination *Zusanli* (ST36) and *Neiguan* (PC6) (but not at either point alone) prevented sensitization of the mesocorticolimbic pathway induced by ethanol in mice and modulated both the expression of the protein homer1A and glutamatergic plasticity [28]. EA (2 Hz) at *Zusanli* (ST36) could reduce voluntary intake of ethanol, but not sucrose, in rats [50] and 100 Hz EA treatment at *Zusanli* (ST36) effectively reduces preference for ethanol and its consumption in rats [49]. In one study, 2 Hz EA at *Zusanli* (ST36) and *Neiguan* (PC6) or 100 Hz EA at *Dazhui* (DU14) and *Baihui* (DU20) inhibited CB1R upregulation in ethanol-withdrawn mice [29]. The behavioral effects of 2 Hz EA at *Dazhui* (DU14) and *Baihui* (DU20), but not 100 Hz EA at *Zusanli* (ST36) and *Neiguan* (PC6), depended on extracellular signal-regulated kinase signaling [30]. See Table 5 for study details.

**Table 5:** Original investigations of acupuncture therapy effects in alcohol-dependent subjects

Publication year; first author		Objectives	Subjects	Stimulated acupoints (acupuncture type, acupoints, frequency)	Assessments	Outcomes
2001	Yoshimoto [86]	To investigate the effect of EA on changes in alcohol-drinking behavior in rats challenged with restriction and immobilization stress	8–12 Male Sprague–Dawley rats	1 Hz and 100 Hz EA at *Zusanli* (ST 36) and *Shenshu* (BL 23) for 10 min, twice a week for 1–3 weeks	Time-access alcohol-drinking behavior, brain dopamine (DA) level	Acupuncture at *Zusanli* (ST 36) was more effective for reducing the increased alcohol-drinking behavior
2002	Bullock [16]	To report the clinical data on the efficacy of acupuncture for alcohol dependence	503 Alcoholics	AA at *Shenmen* (HT7), lung, sympathetic, and liver for 40 min, 6 days a week for 3 weeks	Alcohol use, depression, anxiety, functional status, and preference for therapy	acupuncture was not found to be effective in reduction of alcohol use alone
2002	Karst [39]	To investigate the acupuncture effects on alcohol withdrawal therapy with carbamazepine	34 Alcoholics	*AA at Sympathetic, Shenmen (HT7), kidney, liver, lung,* *Baihui* (GV20), extra1, and *He Gu* (Li4), daily for 10 days	Clinical Institute Withdrawal Assessment (CIWA-Ar-scale)	Acupuncture as an adjunctive treatment to carbamazepine medication shows promise for the treatment of alcohol withdrawal symptoms
2003	Trumpler [74]	To compare auricular laser and needle acupuncture with sham laser stimulation in reducing the duration of alcohol withdrawal	48 alcoholics undergoing alcohol withdrawal with clomethiazole n = 17 laser, n = 15 needle	AA (2-10 out of 24 points) for 30-45 min, laser AA at 24 pints (1 min for each point), 3-4 days	The duration of withdrawal symptoms (nurse-rated scale), duration of sedative prescription	Acupuncture showed no relevant benefit for alcohol withdrawal
2004	Zalewska-Kaszubska [87]	To intensify AA method by additional biostimulation of the whole organism	53 Alcoholics under daily helium–neon laser for neck biostimulation	Laser AA at concha points for 4 periods of ten times applied every 2nd day	The Beck Depression Inventory-Fast Screen (BDI-FS), beta-endorphin plasma concentration by using the radioimmunoassay (RIA)	laser therapy was useful as an adjunct treatment for alcoholism
2005	Kim [40]	To investigate the effects of acupuncture on alcohol withdrawal syndrome (AWS) and Fos-like immunoreactivity (FLI) in the striatum and the nucleus accumbens (NAC) of rats	24 Male Sprague–Dawley rats	Acupuncture at *Zusanli* (ST36) and *Sanyinjiao* (SP6) for 3 days	Alcohol withdrawal syndrome (AWS) and Fos-like immunoreactivity (FLI) in the striatum and the nucleus accumbens (NAC)	acupuncture was useful in the treatment of alcoholism by modulating post-synaptic neural activation in the striatum and NAC
2006	Yoshimoto [85]	To investigate the neuropharmacological mechanisms of oriental acupuncture	24 Male Sprague–Dawley rats, n = 16 acupuncture	unilateral or bilateral acupuncture at *Shenshu* (BU23) acupoint, 60 min	Dopamine (DA) and serotonin (5-HT) contents of the microdialysates in the ACC	Acupuncture was effective for treatment of emotional disorders and laconism by increasing and prolonging the activity of serotonergic neurons
2006	Zhao [89]	To investigate the effects of acupuncture on chronic ethanol-induced changes in extracellular dopamine levels in the nucleus accumbens shell	35 Male Sprague–Dawley rats, n = 21 sham or real acupuncture	Bilateral acupuncture at *Shenmen* (HT7) point or *Neiguan* (PC6) or tail) for 1 min	Extracellular dopamine levels in the nucleus accumbens shell (using in vivo microdialysis in unanesthetized rats)	Acupuncture at HT7 was effective to normalize the release of dopamine in the mesolimbic system following chronic ethanol treatment
2007	Kunz [43]	To compare auricular needle acupuncture with aromatherapy in reducing the duration and severity of symptoms of alcohol withdrawal with carbamazepine, oxcarbazepine, and benzodiazepines	74 alcoholics, n = 36 acupuncture, n = 38 aromatherapy	AA at 5 NADA points, 45 min for 5 days	Alcohol-withdrawal syndrome (AWS scale), subjective visual analog scale of craving and the Self-Assessment Manikin (SAM)	acupuncture was not more effective than the control therapy on alcohol withdrawal symptoms
2008	Overstreet [64]	To investigate the EA effects for reducing voluntary alcohol intake in alcohol-preferring rats	18 inbred alcohol-preferring P rats (IP), n = 9 EA	2 and 100 Hz EA at *Zusanli* (ST36) and *Sanyinjiao* (SP6) for 30 min	Alcohol intake	Acupuncture affected on alcohol intake in the alcohol-dependent IP rats
2009	Dos Santos [28]	To investigate the effects of EA over locomotor sensitization induced by ethanol in mice	12 Male Swiss mice	2 Hz EA at *Zusanli* (ST36) and/or *Neiguan* (PC6) for 10 min	The locomotor activity, the expression of homer1A mRNA assessed by PCR	EA modulated homer1A expression and glutamatergic plasticity
2010	Yang [82]	To evaluate the effects of HT7 acupuncture on VTA GABA neuron excitability, ethanol inhibition of VTA GABA neuron firing rate, and ethanol self-administration	32 Male Wistar rats	2 Hz EA at *Shenmen* (HT7) or *Neiguan* (PC6) for 1 min	Ethanol-Reinforced Responding, VTA GABA Neuron Activity, VTA GABA Neuron Firing Rate	acupuncture reduced ethanol suppression of VTA GABA neuron firing rate, and reduced ethanol self-administration without affecting sucrose consumption
2011	Li [50]	To demonstrate that SD rats escalated their ethanol intake and subsequently developed ethanol dependence under the IE procedure	26 Male Sprague–Dawley rats	2 and 100 HZ EA at *Zusanli* (ST36) for 20 min	Intake of and preference for ethanol	EA treatments decreased the intake of and preference for ethanol, without resulting in a rebound increase in ethanol intake when the EA treatments were terminated
2012	Li [49]	To test the hypothesis that EA suppression on alcohol consumption may be mediated by transcription factors, such as FosB/ΔFosB protein in reward-related brain regions	33 Male Sprague–Dawley rats	2 and 100 HZ EA at *Zusanli* (ST36), 30 min for 6 days	The expression of FosB/ΔFosB in several reward-related brain regions using immunohistochemistry	EA treatment effectively reduced ethanol consumption and preference in rats by down-regulation of FosB/ΔFosB in reward-related brain regions
2012	Escosteguy-Neto [29]	To investigate EA effects during ethanol withdrawal on CB1R immunoreactivity	12 Male Swiss mice	2 and 100 Hz EA at *Zusanli* (ST36)/*Neiguan* (PC6) or *Dazhui* (DU14)/*Baihui* (DU20), 10 min for 4 days	CB1R in the prefrontal cortex, striatum, hippocampus, amygdala and ventral tegmental area	EA inhibited CB1R upregulation which depended on acupoints association and frequency of stimulation
2012	Fallopa [30]	To investigate whether EA reverses locomotor sensitization induced by ethanol is parallel to ERK signaling	12 Male Swiss mice	2 and 100 Hz EA at *Zusanli* (ST36)/*Neiguan* (PC6) or *Dazhui* (DU14)/*Baihui* (DU20), 10 min for 4 days	pERK immune-histochemistry	EA increased CB1R in the prefrontal cortex, striatum, hippocampus, amygdala and ventral tegmental area

Acupoints in NADA protocol are located at (sympathetic: in the deltoid fossa at the junction of the infra-antihelix crus and the medial order of the helix, lung: in the center of the cavum concha, liver: located in the posterior to upper portion of the helix crus, kidney: in the cleft between the upper plateau, and the helix)

### Morphine

Compared with 100 Hz, 2 Hz peripheral electric stimulation (PES) at *Zusanli* (ST36) and *Sanyinjiao* (SP6) inhibited the expression of morphine-induced conditioned place preference (CPP) (see [52] for information on CPP) via activation of opioid receptors [75]. One study found that the release and synthesis of enkephalin in the NAc was accelerated by 2 Hz stimulation of *Zusanli* (ST36) and *Sanyinjiao* (SP6) [53]. In addition, EA suppression of opiate addiction might involve the release of endogenous μ-, δ-, and κ-opioid agonists in the NAc shell [52] and might activate the cannabinoid, endogenous opioid, and dopamine systems to induce CPP in rats [81]. PES (100 Hz) at *Zusanli* (ST36) and *Sanyinjiao* (SP6) activated the suprasegmental δ- and κ-opioid receptors in the central nervous system, which cause the anticraving effects of PES in rats [70]. It was also found that the expression of preproenkephalin and preprodynorphin mRNAs in the NAc was mediated by 2 Hz or 100 Hz PES, with the release of endogenous μ-, δ-, and κ-opioid agonists to suppress morphine-induced CPP [71]. Stimulation at *Zusanli* (ST36) and *Sanyinjiao* (SP6) (100 Hz) for 30 min normalized the activity of ventral tegmental area dopamine neurons [34], downregulated p-cAMP response element binding, and accelerated dynorphin synthesis in the spinal cord [76].

Some research suggests that 2 Hz EA is a potential complementary therapy for improving immune dysfunction in opiate addicts [51] and that 2 Hz or 100 Hz EA facilitates the recovery of male sexual behavior in rats during morphine withdrawal [27]. Thirty minutes of EA of 2 Hz or 100 Hz at *Zusanli* (ST36) and *Sanyinjiao* (SP6) reversed the morphological alterations induced by chronic morphine administration [25]. In addition, by increasing NREM sleep, REM sleep, and total sleep time, EA could be a potential treatment for sleep disturbance during morphine withdrawal [48].

EA at *Shenshu* (BL23) attenuated the expression of the proto-oncogene c-Fos in the central nucleus of the amygdala [54]. Acupuncture at *Shenmen* (HT7) inhibited neurochemical and behavioral sensitization to morphine by decreasing dopamine release in the NAc [41]. Acupuncture at *Shenmen* (HT7) significantly suppressed morphine-induced increase in locomotor activity and Fos expression in the NAc and striatum [45]. Acupuncture at *Yanggu* (SI5) can reduce the reinstatement of morphine-seeking behaviors by mediating the gamma-aminobutyric acid receptor system [46, 47]. See Table 6 for study details.

**Table 6:** Original investigations of acupuncture therapy effects in morphine-dependent subjects

Publication year; first author		Objectives	Subjects	Stimulated acupoints (acupuncture type, acupoints, frequency)	Assessments	Outcomes
2000	Wang [75]	To observe the effect of peripheral electric stimulation (PES) on morphine-induced Conditioned place preference CPP	57/82 Male Sprague–Dawley rats	2 and 100 Hz PES at *Zusanli* (ST36) and *Sanyinjiao* (SP6) for 30 min	Conditioned place preference	2 Hz PES could specifically inhibit the expression of morphine-induced CPP
2003	Shi [70]	To examine the effect of 100 Hz peripheral electric stimulation (PES) on the expression of morphine-induced CPP	48 Male Sprague–Dawley rats	100 Hz PES at *Zusanli* (ST36) and *Sanyinjiao* (SP6), 30 min a day for 3 days	Conditioned place preference	Repeated 100 Hz PES had anti-craving effects by activating supra-segmental δ- and κ-opioid receptors
2004	Shi [71]	To elucidate if preproenkephalin (PPE) and preprodynorphin (PPD) mRNAs in the nucleus accumbens (NAc) play a role in PES suppressing morphine-induced CPP	48 Male Sprague–Dawley rats	2 and 100 Hz PES at Zusanli (ST36) and Sanyinjiao (SP6), 30 min a day for 3 days	Conditioned place preference	PES suppressed both the expression of morphine-induced CPP and the reinstatement of extinguished CPP
2004	Cui [27]	To investigate the effect of EA on the sexual behavior of male rats undergoing morphine withdrawal	41 Male Sprague–Dawley rats	2 and 100 Hz EA at *Zusanli* (ST36) and *Sanyinjiao* (SP6), 30 min a day for 7 days	Total serum testosterone (TST) concentrations	EA facilitated the recovery of male sexual behavior and increased TST concentrations
2005	Kim [41]	To investigate the effect of acupuncture on repeated morphine-induced changes in extracellular dopamine levels	31 Male Sprague–Dawley rats	Acupuncture at *Shenmen* (HT7) for 1 min	Dopamine release in the nucleus accumbens and behavioral hyperactivity	Acupuncture decreased both dopamine release in the nucleus accumbens and behavioral hyperactivity
2005	Liu [54]	To evaluate the effect of EA on morphine withdrawal signs and c-Fos expression of the amygdala	21 Male Sprague–Dawley rats	100 Hz EA at *Shenshu* (BL23) for 30 min	Corticosterone levels and behavioral responses during EA stimulation	EA significantly reduced the signs of morphine withdrawal
2008	Chu [25]	To observe the effect of EA on chronic morphine-induced neuronal morphological changes in the ventral tegmental area (VTA)	12 Male Sprague–Dawley rats	2 and 100 Hz EA at *Zusanli* (ST36) and *Sanyinjiao* (SP6), 30 min for 3 days	The rough endoplasmic reticulum, membrane configuration of the nucleus and mitochondria, and structure of myelin sheath	EA reversed the morphological alterations induced by chronic morphine administration
2009	Hu [34]	To examine alterations in the firing rate of Dopamine neurons in 100 Hz EA treatment	40 Male Sprague–Dawley rats	100 Hz EA at *Zusanli* (ST36) and *Sanyinjiao* (SP6), 30 min for 10 days	Conditioned place preference	EA was effective for the treatment of opiate addiction by normalizing the activity of VTA DA neurons
2010	Liang [53]	To find the Role of enkephalin in the nucleus accumbens mediating the effects of EA	218 Male Sprague–Dawley rats	2 Hz EA at *Zusanli* (ST36) and *Sanyinjiao* (SP6), 30 min for 1-3 days	Conditioned place preference	EA up-regulated the mRNA level of preproenkephalin in the NAc
2010	Lee [45]	To investigate the effect of acupuncture on morphine-induced behavioral sensitization and the neuronal changes in NAc and striatum	14 Male Sprague–Dawley rats	Acupuncture at *Shenmen* (HT7), 1 min for 3 days	Morphine-induced changes of locomotor activity and Fos expression	acupuncture suppressed the morphine-induced increases in the locomotor activity and Fos expression in the NAc and striatum
2011	Li [48]	To observe whether EA could modulate the immune status of morphine dependence and withdrawal mice	40 Male BALB/c mice	2 Hz EA at *Zusanli* (ST36) and *Sanyinjiao* (SP6), 30 min for 5 days	Splenic T Lymphocyte Proliferation, IL-2 Production, CD4^+^/CD8^+^ Ratio	EA raised IL-2 and normalized chronic morphine exposure-induced immune dysfunctions
2011	Li [51]	To investigate the effect of 2 and 100 Hz EA of the sleep disturbance during morphine withdrawal	15 Male Sprague–Dawley rats	2 and 100 Hz EA at *Zusanli* (ST36) and *Sanyinjiao* (SP6), 30 min twice a day for 3 days	Electroencephalogram and electromyogram	EA decreased NREM/REM and total sleep time, while the sleep latency prolonged significantly during acute morphine withdrawal
2011	Wang [76]	To find the optimum protocol for EA effective for alleviating withdrawal syndrome	40 Male Sprague–Dawley rats	100 Hz EA at *Zusanli* (ST36) and *Sanyinjiao* (SP6) for 30 min	Conditioned place preference	EA down-regulated of p-CREB and accelerated of dynorphin synthesis in spinal cord
2011	Xia [81]	To investigate whether EA by itself will produce some rewarding effect	44 Male Sprague–Dawley rats	2 Hz EA at *Zusanli* (ST36) and *Sanyinjiao* (SP6) for 30 min	Conditioned place preference	EA was capable of inducing CPP in the rat via the activation of the endogenous opioid-, cannabinoid- and dopamine-systems
2012	Lee [47]	To investigate the role of acupuncture in the reinstatement of morphine seeking	15 Male Sprague–Dawley rats	Acupuncture, *Yanggu* (SI5) or *Yangxi* (LI5) for 1 min	Morphine reinstatement	acupuncture attenuated the reinstatement of morphine seeking behavior by blocking the GABA receptor antagonists
2013	Lee [46]	To investigate whether acupuncture could suppress the reinstatement of morphine-seeking behavior	28 Male Sprague–Dawley rats	Acupuncture, *Yanggu* (SI5) or *Yangxi* (LI5) or *Zusanli* (ST36) for 1 min	Morphine-seeking behavior	acupuncture suppressed morphine injection perfectly

Acupoints in NADA protocol are located at (sympathetic: in the deltoid fossa at the junction of the infra-antihelix crus and the medial order of the helix, lung: in the center of the cavum concha, liver: located in the posterior to upper portion of the helix crus, kidney: in the cleft between the upper plateau, and the helix)

### Other substances

Studies of methamphetamine, cannabis, illicit/psychoactive drugs, and polydrug users are shown in Table 7. Twelve studies used the NADA 5-point protocol and AA as their treatment method. The findings indicated that people dependent on drugs preferred acupuncture treatment [9], which was associated with a decrease in psychological distress [12] and an increase in confidence [14], but showed no efficacy for drug consumption and withdrawal symptoms [9, 12–14]. However, the conflicting nature of the research findings remains a controversial issue. Although there was evidence against the effectiveness of acupuncture in drug addiction treatment [7, 35, 36], recent studies have shown an effect for AA [23, 24, 26, 68, 73] and transcutaneous electric acupoint stimulation [66] per se or as adjunct treatments. Issues of safety and placebo effects suggest the need for further research [26, 35, 36, 66]. See Table 7 for study details.

**Table 7:** Original investigations of acupuncture therapy effects in poly-drug and other substance abusers (*NA* not available)

Publication year; first author		Objectives	Subjects	Stimulated acupoints (acupuncture type, acupoints, frequency)	Assessments	Outcomes
2000	Russell [68]	To compare the behavior of addicts in a treatment center with archived information from no-acupuncture (NA) patients	86 Patients (Methamphetamine was the primary drug of choice for 44)	AA at 5 NADA points, 45 min for 9 weeks	Program retention, new arrests incurred, drug-positive urinalysis results, and number of days needed to progress from entry level to secondary level treatment	Acupuncture improved program retention up to 30 days among methamphetamine-addicted patients
2000	Song Bernstein [14]	To explore the meaning of substance abusers’ experience while receiving acupuncture as a part of the treatment for substance dependence	8 Human Subjects	AA, (NA), once a week	Interviews, researcher’s field notes, and demographic data obtained from the participants’ medical records	Acupuncture caused anticipation of pain, apprehension concerning a new experience, mood elevation, inability to describe the experience, physical sensation, relaxation, and improved sleep
2004	Berman [13]	To compare the experimental NADA-Acudetox protocol with a non-specific helix control protocol in a randomized trial	174 Inmates	Auricular acupuncture at 5 NADA points, 40 min for 4 weeks	A simple drug use questionnaire, the Acupuncture Treatment Assessment Scale (ATAS), a Swedish research version of the Symptom Check List 90	Acupuncture had no significant efficiency over the placebo
2005	Janssen [36]	To examine the utility of acupuncture treatment in reducing substance use in the marginalized, transient population	261 Humans	AA at 5 NADA points for 40 min	Questionnaire, Drug use symptomatology, severity of withdrawal symptoms	Acupuncture caused reduction in overall use of substances and decrease in intensity of withdrawal symptoms
2006	Tian [73]	To examine the efficacy of AA in addition to usual care in substance abuse treatment	17 Humans	AA at 5-points NADA, once a week for 6 consecutive weeks	The Hopkins Symptom Checklist (SCL-20) depression scale, brief substance craving scale	there was a positive response to the specific auricular acupressure treatment on psychological distress, craving, and drug/alcohol use measures
2007	Courbasson and Christine [26]	To evaluate the benefits of adding AA to a 21-day outpatient structured psychoeducational treatment program	185 Women with concurrent substance use problems, anxiety, and depression	AA at 5 points NADA for 45 min, 3 times a week	Physiological cravings for substances, depression, and anxiety	AA as an adjunct therapy to a comprehensive psychoeducational treatment was effective and more viable treatment alternative to anxiolytics
2009	Ashton [9]	To describe the characteristics of clients choosing AA or counseling to treat dependence at a UK self-referral center	162 Humans, n = 36 acupuncture, n = 126 counselling	AA at 5-points NADA, 45 min, once a week for 11 weeks	Psychometric variables (anxiety, depression, dependence severity, readiness to change), and alcohol and drug consumption	Acupuncture was preferred by clients and follow up assessments showed a significant decrease in psychological distress and reduction of alcohol consumption
2014, 2010	Chang and Bei-Hung [23, 24]	Examine the effects of acupuncture and related response (RR) on reducing craving	23 Acupuncture, 23 RR, 21 controls (homeless military veterans)	AA at 5-points NADA, 45 min twice a week	Degree of craving and anxiety levels	acupuncture and the relaxation reduced craving and anxiety levels
2011	Black, S. [7]	To test the hypothesis if AA reduces the anxiety associated with withdrawal from psychoactive drugs.	101 Patients recruited from an addiction treatment service	AA at 5-points NADA 45 min for 3 days	Anxiety state by using a pretest–posttest treatment design	The NADA protocol was not more effective than sham or treatment setting control in reducing anxiety
2012	Janssen [35]	To test the ability of maternal acupuncture treatment among mothers who use illicit drugs to reduce the frequency and severity of withdrawal symptoms among their newborns	50 Women using acupuncture, 39 women standard care	AA at 5-points NADA for 45 min	Days of neonatal morphine treatment for symptoms of neonatal withdrawal. Neonatal outcomes included admission to a neonatal ICU and transfer to foster care	length of treatment for neonatal abstinence syndrome showed no efficiency of acupuncture
2012	Penetar [66]	To investigate the effects of TEAS on drug addiction	9 Cocaine-dependent, 11 Cannabis-dependent	2 and 100 Hz at *Neiguan* (PC6)/*Waiguan* (TH5) and *Hegu* (LI4)/*Laogong* (PC8) stimulation, Twice-daily 30-minute sessions of for 3.5 days	Drug use and drug cravings, cue-induced craving EEG evaluation, and P300 ERP	TEAS did not reduce drug use or drug cravings, or alter the ERP peak voltage or latency but modulated several self reported measures of mood and anxiety
2014	Bergdah [12]	To describe patients’ experiences of receiving AA during protracted withdrawal	15 Human subjects	AA at 5-points NADA 40 min, twice a week for 5 weeks	Interview	AA reinforced sense of relaxation and well-being, peacefulness and harmony, and new behaviors

Acupoints in NADA protocol are located at (sympathetic: in the deltoid fossa at the junction of the infra-antihelix crus and the medial order of the helix, lung: in the center of the cavum concha, liver: located in the posterior to upper portion of the helix crus, kidney: in the cleft between the upper plateau, and the helix)

## Conclusion

AA and NADA protocols failed to show a strong therapeutic effect for cocaine, nicotine, and alcohol addiction treatment. However, some studies discussed here indicate that acupuncture at *Shenmen* (HT7), *Zusanli* (ST36), and *Sanyinjiao* (SP6) acupoints can affect drug-induced physiological activities.
